# Hirntod erleben, ärztliche Sichtweise – ein phänomenologischer Ansatz

**DOI:** 10.1007/s00063-022-00905-9

**Published:** 2022-03-08

**Authors:** Sabine Drexler, Erik Farin-Glattacker, Christiane Kugler

**Affiliations:** 1 Klinik für Neurochirurgie, Klinik für Neurologie und Neurophysiologie, Universitätsklinikum Freiburg, Breisacher Str. 64, 79106 Freiburg, Deutschland; 2 Fakultät für Gesundheit, Department Pflegewissenschaft, Universität Witten/Herdecke, Witten, Deutschland; 3 Insititut für Medizinische Biometrie und Statistik, Sektion Versorgungsforschung und Rehabilitationsforschung, Universitätsklinikum Freiburg, Freiburg, Deutschland; 4 Institut für Pflegewissenschaft, Medizinische Fakultät, Albert-Ludwigs-Universität Freiburg, Freiburg, Deutschland

**Keywords:** Qualitative Forschung, Interview, Interpretative Phänomenologie, Sterben, Übergang, Qualitative research, Interview, Interpretative phenomenology, Dying, Transition

## Abstract

**Ziel:**

Ziel der vorliegenden Studie war die Untersuchung des Erlebens von Ärzten bei der Betreuung von (potenziell) hirntoten Patienten.

**Methodik:**

Durchgeführt wurden episodische Interviews. Die Auswertung fand in Anlehnung an die interpretative Phänomenologie nach Benner statt.

**Ergebnisse:**

Elf Ärzte haben an den Interviews teilgenommen. Folgende Phänomene konnten aus den Daten gewonnen werden: 1) Begegnungen mit den Angehörigen, 2) Angehörigen den Hirntod begreiflich machen, 3) Hirntod ist Tod, 4) Erleben der Pflegenden und 5) Belastungen.

**Schlussfolgerung:**

Die Ergebnisse zeigen die Komplexität, die bei der Betreuung eines (potenziell) hirntoten Patienten aus Sicht der Ärzte wahrgenommen wird. Der Übergang vom Erhalt des Lebens des Patienten zum Organerhalten oder zum Abstellen der Geräte und die dazugehörige Aufklärung der Angehörigen erfordern hohe Kompetenzen der Ärzte. Symptome der Patienten geben den Ärzten klare diagnostische Vorgehensweisen, bestehende Unklarheiten im (Behandlungs‑)Prozess sollten behoben werden.

## Abstract (en)

**Aim:**

The aim of the study was to examine how physicians experience caring for (potentially) brain-dead patients.

**Methods:**

Episodic interviews were conducted. Their evaluation was based on Benner’s interpretative phenomenology.

**Results:**

Eleven physicians were interviewed. The following phenomena were extracted from the data: (1) encounter with the relatives, (2) making relatives understand brain death, (3) brain death is death, (4) experience of the nurses and (5) burdens.

**Conclusion:**

The results show the complexity that is perceived by physicians when caring for a (potentially) brain-dead patient. The transition from saving the patient’s life to preserving organs or switching off the equipment and informing the relatives requires a high level of expertise on the part of the physicians. The patients’ symptoms provide the physicians with clear diagnostic procedures and any remaining ambiguities in the (treatment) process should be resolved.

## Einleitung

Die Betreuung von (potenziell) hirntoten Patienten auf Intensivstationen ist eine Herausforderung, die aus Sicht der Ärzte bisher kaum untersucht ist. Zum subjektiven Erleben dieser besonderen Situation gibt es daher wenige Daten. Dieser Artikel möchte die berichteten, erlebten Situationen der interviewten Ärzte verstehen.

Es gibt zu diesem Thema aus Deutschland lediglich quantitative Studien, z. B. über den Organspendeprozess [7] oder zur postmortalen Organspende [16]. In einer ebenfalls quantitativen Studie aus Frankreich wurden Ärzte zur Organspende oder verwandten Tätigkeiten befragt [9]. In allen 3 Befragungen sind die Ärzte gegenüber der Organspende eher positiv oder neutral gestimmt (90 % [7], 84 % [16] und 84 % [9]), allerdings werden hier die Einstellungen zur Organspende abgefragt und nicht das Erleben der Betreuung eines (potenziell) hirntoten Menschen. In einer qualitativen Befragung aus Brasilien wird die Betreuung eines hirntoten Organspenders mit der eines lebenden Intensivpatienten aus Sicht der Ärzte gleichgesetzt. Die Aufklärung der Angehörigen bei hirntoten Patienten wird als schwierig beschrieben, da diese Patienten nicht tot aussehen. Die teilnehmenden Ärzte fühlten sich für die Aufklärungsgespräche nicht vorbereitet [15].

Ziel der vorliegenden Studie ist es herauszufinden, wie Ärzte in Deutschland die Betreuung von (potenziell) hirntoten Patienten erleben. Mit dem Begriff „potenziell“ ist der Verlauf der Patienten auf einer Intensivstation gemeint. Hier wird mit einer möglichen kurativen Behandlung begonnen, dann kommt die Phase des möglichen Hirntods mit ggf. der Hirntoddiagnostik und der Entscheidungsfindung.

Die durchgeführten Interviews mit den Ärzten waren Gegenstand einer größeren Studie, in der Pflegende, Ärzte und Angehörige von (potenziell) hirntoten Patienten zu ihrem Erleben zur Betreuung von (potenziell) Hirntoten interviewt wurden, um aus den Ergebnissen Praxisempfehlungen für den Versorgungsalltag zu erstellen [5]. Die Durchführung der Studie wurde durch die Ethikkommission (EK) der Albert-Ludwigs-Universität Freiburg genehmigt (EK-Freiburg 87/16) und ist im Deutschen Register Klinischer Studien registriert (DRKS00010420).

## Methodik

Es wurde ein qualitatives Verfahren gewählt, um die erlebten Situationen, die die teilnehmenden Ärzte beschrieben haben, zu verstehen. Hierbei können die Erlebnisse der Ärzte in ihren Sichtweisen gedeutet werden. Die Fragen sind offen gestellt und eine Standardisierung, wie bei einem Fragebogen, ist nicht möglich [10]. Zur Datenerhebung wurden episodische Interviews ausgewählt [6]. Hierbei wird über Erzählungen (narrativ) das Erlebte erzählt und über anschließendes konkretes Nachfragen das semantische Wissen generiert. Das bedeutet, dass nach der erlebten, erzählten Situation die Erlebnisse auf weitere Situationen generalisiert werden [6]. Die gewonnenen Daten wurden in Anlehnung an die interpretative Phänomenologie von Benner ausgewertet [1]. Hierbei kommt es zu einer detaillierten Interpretation eines Phänomens, wie es von den Teilnehmenden erlebt wird [11]. Grundlage ist die phänomenologische Forschung von Heidegger [8], das Vorverständnis der Erstautorin fließt ebenfalls mit ein [1], sie ist selbst Fachkrankenschwester für Intensivpflege und Anästhesie.

Während der Interpretationen entsteht ein Wechsel zwischen dem Vorverständnis der Erstautorin und dem Verstehen der Interviews [17]. Es wird eine „thematische Analyse“ durchgeführt, „Paradigma Fälle“ und „Musterbeispiele“ werden identifiziert [1].

Die thematische Analyse der Interviews wurde teilweise durch 2 Forschende durchgeführt, die analysierten Themen von der Erstautorin verglichen und Themen identifiziert. Im nächsten Schritt fand die vertiefte Analyse jedes einzelnen Interviews statt. Hierbei wurden die Interviews auf Grundlage der Fragestellung zusammengefasst, um ein vertieftes Verständnis der zentralen Belange zu bekommen (Tab. 1; [2]). Die Ergebnisse beider Schritte wurden verglichen, um die erlebten Situationen zu verstehen. Bei schwierigen oder besonderen Themen konnten Teilstücke der originalen Interviewtexte oder Interpretationen mit qualitativ Forschenden in Arbeitsgruppen diskutiert werden.

**Table 1:** 

Zentrale Themen	Verstehen der Erlebnisse der Ärzte
Handlungssicherheit	Trennung lebender Patient – hirntoter Patient, tot/Tod, Endgültigkeit, Leitlinien vorhanden, klare Vorgaben
	Zusammenarbeit mit den Pflegenden, weiteren Akteuren etc.
Aufklärungsgespräche	Hirntod den Angehörigen begreiflich machen, Ausbildung in der Gesprächsführung, bessere Dokumentation der besprochenen Dinge, Begrifflichkeiten definieren
	Aufklärungsgespräche: Fachärzte wollen Assistenzärzte einarbeiten, Assistenzärzte denken nicht, dass eine Einarbeitung durch Fachärzte funktioniert, sie würden einen erfahrenen Kollegen bevorzugen
	Hierarchie, am Wochenende geht es auch alleine? – keine Hierarchie?
	Reingeschmissen werden – Gesprächsführung an der Universität lernen bringt nichts, wird aber angewandt – Widerspruch
Haltung	Verarbeitung der Situation, Therapie und Aushalten bis eine Entscheidung getroffen ist; Schuldgefühle – Versagen – Hirntod – ärztliches Versagen
Fehlende Rahmenbedingungen	Minderjährige Kinder als Besucher der Intensivstation; Bettenpolitik, Sterbebegleitung

Teilnahmekriterien waren: approbierter Arzt, der bereits erwachsene hirntote Patienten betreut hat.

Das Forschungsvorhaben wurde an leitende Ärzte unterschiedlicher Fachabteilungen mit Intensiveinheiten an einem Universitätsklinikum in Deutschland versendet mit der Bitte um Weiterleitung an die entsprechende Zielgruppe. Die Ärzte, die an einem Interview teilnehmen wollten, konnten sich bei der Erstautorin melden. Nachdem sie über das Forschungsvorhaben umfassend aufgeklärt waren („informed consent“) und schriftlich einwilligten, wurde ein Termin für das Interview vereinbart. Alle Interviews fanden in Räumlichkeiten des Universitätsklinikums statt und wurden von der Erstautorin selbst durchgeführt.

Der Interviewleitfaden wurde von einer Vorstudie zum Erleben der Pflegenden in der Betreuung von (potenziell) hirntoten Patienten für Ärzte angepasst [4].

Die Interviews wurden digital aufgezeichnet, pseudonymisiert, von einem Unternehmen wörtlich (ohne Dialekt) transkribiert und für die erste thematische Analyse in das Programm MAXQDA (Verbi, Release 12.3.0) eingepflegt. Der vorliegende Artikel wurde nach der Erstellung durch die Erstautorin hinsichtlich der Qualität mit der Checkliste COREQ [18] überprüft.

## Ergebnisse

Zwischen Mai 2016 und August 2017 wurden von der Erstautorin 11 Interviews mit Ärzten (9-mal männlich, 2‑mal weiblich) mit unterschiedlichen Berufserfahrungen auf einer Intensivstation (zwischen 5 Monaten und > 10 Jahren) und -qualifikationen (Assistenzärzte, Fachärzte, leitende Ärzte) durchgeführt. Die Interviews dauerten in Summe 263,57 min, was einem Mittelwert von 24 min entsprach (von 9 min 21 s bis 31 min 36 s).

Aus den Hauptthemen und den zentralen Belangen sind die folgenden Phänomene gedeutet worden.

## Begegnungen mit den Angehörigen

Die Angehörigen nehmen in den gewonnenen Interviewdaten eine bedeutende Rolle ein. Die geschilderten Erlebnisse beziehen sich größtenteils auf die Erlebnisse mit den Angehörigen. Manche der interviewten Ärzte wirken unsicher in Bezug auf das, was sie den Angehörigen von Hirntoten zumuten können. Es ist ihnen unklar, wann sich die Angehörigen von dem hirntoten Patienten verabschieden sollten. In einem Interview wird die Einbindung einer Angehörigen in die Extubation des Hirntoten sehr positiv erlebt.

„*Und haben wir dann auch zusammen gemacht, mit einer sehr erfahrenen Pflege, was sehr gut war. Ja. Und deswegen/das ist so das, was ich noch so in Erinnerung habe. Und ich sehe diese Frau heute noch vor mir, wie sie an diesem Bett sitzt, nachdem die Maschinen aus waren … hat sich auch bedankt bei uns, also bei mir und der Pflege*.“ (A11)

Den interviewten Ärzten ist es wichtig, dass sich die Angehörigen keine Vorwürfe machen, nicht alles getan zu haben. Sie müssen das sichere Gefühl vermittelt bekommen, dass es keine Therapieoption mehr gab. Gerade wenn die Hirntoten die eigenen Kinder sind (auch Volljährige sind noch Kinder der Eltern) oder wenn die Angehörigen minderjährige Kinder sind, treten zusätzliche Herausforderungen auf.

„*Wir Assistenten mussten ziemlich dafür kämpfen, dass dieses Kind dann mit auf Station durfte, weil eigentlich sind die Kinder ja verboten*.“ (A5)

Hierbei spielen die eigenen Haltungen der interviewten Ärzte eine wichtige Rolle. Es wird neben Empathie auch Durchhaltevermögen benötigt, um den Angehörigen die Situation erträglicher zu gestalten und Besonderes möglich zu machen. Dennoch müssen sie sich ausreichend von den erlebten Situationen abgrenzen.

„*Schlimm sind immer nur die Angehörigen, insbesondere, wenn man in irgendeiner Form sich mit denen identifizieren kann*.“ (A3)

## Angehörigen den Hirntod begreiflich machen

Ein in mehreren Interviews relevanter Aspekt ist das Wissen, *„wo man die Angehörigen abholen kann“* (A1). Dies bezieht sich auf den Wissens- und Informationsstand der Angehörigen über den Patienten. Die interviewten Ärzte erlebten immer wieder in den Gesprächen, dass die Angehörigen bei der Aufklärung über die Diagnose Hirntod oder die Möglichkeit des Hirntods die Situation nicht wahrhaben können. Es ist für die Angehörigen unbegreiflich, dass der Patient versterben wird oder verstorben ist. Besonders schwierig scheint es zu sein, wenn zuerst eine kurative Therapie eingeleitet und die Angehörigen hierzu aufgeklärt wurden. Einige interviewte Ärzte erlebten, dass Informationen wie z. B. *„jetzt haben wir operiert“* (A9) von den Angehörigen so verstanden werden, dass nun alles wieder gut ist. Daher ist es den interviewten Ärzten wichtig: …

„*Wenn jetzt der Hirntod schon relativ feststeht, wenn eigentlich von (.) ne, dann kann man den Angehörigen das auch gleich so sagen. Man muss überhaupt keinen Spielraum mehr für irgendwelche Alternativen lassen*.“ (A5)

## Hirntod ist Tod

Die interviewten Ärzte berichten von eindeutigen Maßnahmen, die, je nach klinischem Befund der Patienten, eingeleitet werden. Auf Basis dieser Befunde und der Informationen des Patienten oder von den Angehörigen werden die weiteren Entscheidungen getroffen.

„*Dann weiß man von der Geschichte her, der ist schon sofort beim Umkippen beim Zusammenbrechen, beidseits [Pupillen] weit gewesen, Hirn schon Einklemmung. Das ist jetzt anderthalb Stunden her. Man kann auch eine EVD [externe Ventrikeldrainage] legen, aber wie gesagt, man sieht einen Wahnsinnshirndruck, beidseits weit geblieben. Das war es*.“ (A1)

Dieses Vorgehen macht es den interviewten Ärzten leichter, da sie genau wissen was wann durchzuführen ist, auch wenn einige von ihnen mit den Vorgaben und Leitlinien unzufrieden sind.

„*… die Diagnostik, die Einleitung der Diagnostik, grundsätzlich immer verzögert ist, weil man die Medikamentenspiegel braucht. Das dauert immer, dadurch verzögert sich das Ganze, das ist schlecht für die Angehörigen und auch schlecht für uns, weil man im Prinzip an dem Patienten auch nichts mehr machen*.“ (A6)

Sobald die Diagnostik durchgeführt und die Entscheidungen getroffen wurden, stellt dies für die interviewten Ärzte eine Erleichterung dar – egal für welchen Weg sich entschieden wird. Sie sehen in der klinischen Behandlung eines Hirntoten keinen Unterschied zu lebenden Patienten, die entsprechenden Untersuchungen und Behandlungen werden durchgeführt.

„*Es macht da im Prinzip keinen großen Unterschied, ne, ob man jetzt einen Patienten betreut, der im tiefen Koma liegt oder der hirntot ist. Weil man das von außen nicht sieht. Man weiß es nur. (…) Eine Sterbebegleitung in dem Sinne kann es ja gar nicht geben, weil der Patient ist ja schon tot*.“ (A6)

Obwohl in der medizinischen Versorgung kein Unterschied gemacht wird, ist für die interviewten Ärzte der hirntote Patient dennoch ein Toter. Es bestehen keine Zweifel oder Unsicherheiten an der Diagnose Hirntod:

„*… Der Patient ist tot, hirntot ist tot. Die Tatsache, dass der noch am Beatmungsgerät hängt, macht ihn nicht weniger tot für mich*.“ (A3)

„*… Das ist ja nur die Maschinen, die diesen Körper noch am Leben erhalten*.“ (A11)

In der Zeit, in der die Patienten bereits als hirntot diagnostiziert wurden, es jedoch noch keine finale Entscheidung gibt, in welche Richtung (Abstellen der Geräte oder Einleiten der Organspende) es geht, werden Maßnahmen eingeleitet, damit die Organe erhalten bleiben.

„*… Fast ein bisschen nervig, ja, dass hier ja zum Teil schwerkranke lebende Menschen leben, also auch hirnlebende Menschen, um die man sich kümmern soll. Aber dann der einzig Hirntote, der auf der Station liegt, am Schluss noch am meisten Arbeit macht (…). Den Körper muss man am Leben erhalten irgendwie, ja*.“ (A4)

Die Möglichkeit einer Organspende wird bei den interviewten Ärzten überwiegend positiv erlebt. Die hoffnungslose Situation kann durch eine mögliche Organspende einen neuen Sinn bekommen.

„*Und das erste Mal, dass ich einen Rückmeldebogen bekommen habe. Also einen Brief kriegt man ja immer von Organ ist da! Organ ist da und so! Das war schon sehr berührend muss ich sagen. Das war sehr, das war sehr, sehr ergreifend*.“ (A1)

## Erleben der Pflegenden

Die interviewten Ärzte erleben, dass die Pflegenden in der Regel keinen Unterschied in der Pflege und Betreuung von hirntoten Patienten oder komatösen/sedierten Patienten machen. Erfahrene Pflegende werden bei Unklarheiten, Unsicherheiten, als Stütze bei schwierigen Tätigkeiten und als Feedbackperson für das eigene Vorgehen von unerfahrenen Ärzten mit einbezogen.

„*… Und ich irgendwie so von ihr das Feedback bekommen habe, du machst das gerade richtig. Und wir machen das gerade alles korrekt so. Und sie hat die Maschinen ausgemacht dann aber auch. Was mir sehr recht war für das erste Mal*.“ (A11)

Diese Unterstützung gilt für die meisten interviewten Ärzte jedoch nur bis zu dem Moment, an dem Entscheidungen getroffen werden müssen. Hier wird deutlich, dass erfahrene Pflegende für Ratschläge willkommen sind, aber die Entscheidungsverantwortung bei den Ärzten liegt.

„*… Äh und jetzt für weitere Therapiemaßnahmen, ähm hätte ich jetzt/hatte ich jetzt nicht so den Eindruck, dass man noch die Pflege mit einbezieht*.“ (A10)

## Belastungen

Die Wahrnehmung, wie die interviewten Ärzte für die Aufklärungsgespräche mit den Angehörigen vorbereitet werden, ist unterschiedlich. Von den meisten wird erlebt:

„*… Man kann das schon so pauschal sagen, [man wird] in seiner ärztlichen Laufbahn halt in die Dinge reingeschmissen. Irgendwann mal steht man halt vor dem ersten toten Patient und hat die ersten weinenden Angehörigen um sich rum und muss dann irgendwie mit denen klarkommen*.“ (A4)

Durch die unterschiedlichen Besetzungen in den Schichten der Ärzte wird erlebt, dass nicht immer Fachärzte vor Ort sind, um Assistenzärzte anzulernen oder auch um sie zu unterstützen.

„*… Aber entscheiden tut das letztendlich in aller Regel der Oberarzt, es sei denn, es ist das Wochenende. Dann fällt eine wesentlich größere Last auf uns, weil wir letztendlich die Gesamtsituation zusammentragen müssen sozusagen*.“ (A3)

Weiter wird das Verhalten, das bei den Angehörigen in den Akutsituationen auftritt, extrem erlebt. Es ist nicht vorhersehbar und die Angehörigen müssen individuell abgefangen werden.

„*Aber die Frau wollte es erst nicht verstehen, was/ich wollte sie langsam drauf hinführen, aber sie hat mich dann sozusagen, ja, fast angeschrien, so nach dem Motto: Was ist jetzt los? Und dann habe ich halt letztendlich gesagt: „Ihr Mann wird sterben, ja? Und zwar ziemlich bald“. Und dann ist sie natürlich völlig zusammengebrochen*.“ (A2)

Um die Situation der Angehörigen zu verbessern, wünschen sich die teilnehmenden Ärzte einen Raum, in dem sie ungestört Aufklärungsgespräche durchführen können. Weiter bemängeln sie, dass es keine Räumlichkeiten gibt, in denen sich Angehörige nach einer Organexplantation von dem Verstorbenen verabschieden können. Entsprechend könnten die baulichen Gegebenheiten einer Intensivstation optimiert werden.

„*Dass man Einzelboxen hat, (…) wo die Angehörigen da sind und Abschied nehmen wollen*.“ (A6)

Außerdem sollte ein eindeutiges Vorgehen bei der Dokumentation der Inhalte bei den Aufklärungsgesprächen beschrieben sein, damit diensthabende Ärzte wissen, was den Angehörigen bereits gesagt wurde. Als schwierig wird erlebt, dass verschiedene Begrifflichkeiten, die auf Intensivstationen im Zusammenhang mit einer infausten Prognose verwendet werden, unterschiedlich definiert sind:

„*… Das Hauptproblem, was ich in letzter Zeit hatte, ist, dass die Terminologie unterschiedlich verwendet wird, was man jetzt unter Therapie Einfrieren versus Minimalisierung alles versteht und was man dann auch alles machen kann oder soll. Aber das, das ist letztlich ein, ja, ein Kommunikationsproblem, wo ich mir fast wünschen würde, dass das mal jemand aufschreibt, weil das dann irgendwie immer alles anders ist, wenn/weil man ja natürlich rechtlich in so einer schwierigen Situation ist, wenn man den Dienst übernimmt. (…) Was bedeutet was? Was bedeutet Einfrieren? Was bedeutet Minimalisierung überhaupt?*“ (A3)

## Diskussion

Die Ergebnisse dieser Studie verdeutlichen die Besonderheiten bei der Betreuung von (potenziell) hirntoten Menschen. In den erlebten Situationen der interviewten Ärzte sticht die gegebene Handlungssicherheit bei den diagnostischen Maßnahmen hervor, die durch Leitlinien [3] vorgegeben sind.

Anders wird dies in einer Studie aus Brasilien beschrieben. Hier erleben die interviewten Ärzte unterschiedliche Vorgehensweisen bei der Hirntoddiagnostik, was bei ihnen zu Bedenken und Unsicherheiten führt [15].

Trotz der beschriebenen Handlungssicherheit bei der Diagnostik werden besondere Situationen schwierig erlebt. Dies gilt vor allem für die Aufklärungsgespräche mit den Angehörigen. Hier erleben die interviewten Ärzte eine oftmals unvorbereitete Situation und sie müssen unter diesen erschwerten Bedingungen den Angehörigen den Hirntod des Verstorbenen begreiflich machen. Die Schwierigkeit des „Begreiflichmachens“ wird auch bei Orøy et al. (2015 [14]) beschrieben, die hirntoten Patienten sehen für die Angehörigen wie alle anderen Intensivpatienten aus. Bei der Aufklärung der Angehörigen versuchen die interviewten Ärzte die Angehörigen ebenso offen wie möglich aufzuklären, damit sie für alle Eventualitäten vorbereitet sind. Die Ergebnisse der Diagnostik werden den Angehörigen erst bekannt gegeben, wenn es keine Zweifel mehr gibt [14]. Die interviewten Ärzte unserer Studie beschreiben ein ähnliches Vorgehen. Sie versuchen frühzeitig die Schwere der Erkrankung zu kommunizieren und sobald der Hirntod feststeht, werden die Prognose und deren Möglichkeiten vermittelt.

Die Sterbebegleitung bei dieser Patientengruppe wird anders erlebt, da bei den hirntoten Patienten die Therapie des Körpers weiter durchgeführt wird. Den interviewten Ärzten ist es manchmal unklar, was Angehörige wollen und was ihnen zumutbar ist. Jedoch sind sie sich sicher, dass es wichtig ist, den Angehörigen Sicherheit zu vermitteln, wenn sich die Angehörigen von dem hirntoten Patienten verabschieden. Orøy et al. (2013) beschrieben ebenfalls die Schwierigkeit, die Angehörigen zu begleiten, wenn sie sich vom „lebenden Körper“ verabschieden sollen [13]. Die anschließende Frage nach der Organspende wird als eine besondere Situation beschrieben [13–15].

In den berichteten, erlebten Situationen sind die Pflegenden den Ärzten in der Begleitung der Therapie eine Unterstützung und werden für ihre Fachkompetenz sehr geschätzt. Jedoch halten sich die Pflegenden aus den Therapieentscheidungen heraus und einer der interviewten Ärzte hat das Gefühl, dass die Pflegenden hierzu auch nicht eingebunden werden möchten. Im Gegensatz hierzu werden in Norwegen die Pflegenden teilweise in die Entscheidungen eingebunden [14]. Pflegende sind bei den Aufklärungsgesprächen dabei und bleiben anschießend bei den Angehörigen [13].

Kritisch sehen die interviewten Ärzte die baulichen Gegebenheiten der Intensivstationen (Mehrbettzimmer), den mangelnden Platz und damit verbunden das Fehlen eines ruhigen Orts, um die Gespräche mit den Angehörigen zu führen, bzw. der Möglichkeit für die Angehörigen, sich von ihrem Verstorbenen zu verabschieden. Weiter sind unterschiedliche Begrifflichkeiten (*„Einfrieren“, „Minimalisierung“ *A3) im Gebrauch, die nicht einheitlich definiert sind, jedoch Unterschiede in der Behandlung des Patienten machen können. Unklar hierbei ist, warum die in der Literatur beschriebenen Definitionen und Dokumentationsmöglichkeiten [12] nicht genutzt werden. In den Ergebnissen von Orøy et al. (2015) ist zu Unklarheiten in den Begrifflichkeiten nichts beschrieben [14].

Die Ergebnisse der durchgeführten und analysierten Interviews ermöglichen einen vertieften Einblick in die erlebte Welt der interviewten Ärzte mit (potenziell) hirntoten Patienten.

## Limitationen

Da es sich um eine qualitative, phänomenologische Studie handelt, sind die Ergebnisse nur auf die interviewten Ärzte des hier einbezogenen Universitätsklinikums zu begrenzen. Um die Ergebnisse zu vertiefen, müssten:

- mehrere Kliniken mit unterschiedlichen Größen und Schwerpunkten eingebunden werden;
- mehrere Forscher hinzugezogen werden, um getrennt voneinander die Interpretationen durchzuführen;
- die Ergebnisse mit der Unterstützung von Fragebögen o. ä. quantifiziert werden.

Eine weitere Limitation ist, dass die Erstautorin zu dem Zeitpunkt der Durchführung der Interviews im gleichen Universitätsklinikum arbeitet, in dem die interviewten Ärzte arbeiten.

## Ausblick

Potenziell hirntote Patienten und hirntote Patienten wird es immer wieder auf den Intensivstationen geben, daher ist es erforderlich, dass die Ärzte besser auf die damit verbundenen Situationen vorbereitet werden sollten.

Daher ist in einem weiteren Schritt geplant, auf Grundlage der Ergebnisse Praxistipps für die Behandlungsteams auf den Intensivstationen zu erstellen. Diese sollen allgemein formuliert werden, damit sie an die vorhandenen Gegebenheiten angepasst und implementiert werden können.

## Fazit für die Praxis

- Die teilnehmenden Ärzte sehen den Hirntoten als tot an.
- Organspende ist aus Sicht der teilnehmenden Ärzte eine sinnvolle Option.
- Aufklärungsgespräche der Angehörigen sind anspruchsvoll und könnten interprofessionell gestaltet werden.
- Wenn keine Handlungsleitfäden für bestimmte Situationen vorhanden sind, ist das Vorgehen schwierig.
- Begrifflichkeiten, die für Therapieentscheidungen relevant sind (z. B. Einfrieren etc.), müssen definiert werden.
